# Metformin-Sensitized Chemotherapy of Docetaxel Nanoemulsions Based on a Sequential Administration

**DOI:** 10.3390/pharmaceutics17070812

**Published:** 2025-06-23

**Authors:** Junlei Zhang, Jiapeng Mao, Yilong Hu, Xingze Huang, Jian You, Lihua Luo

**Affiliations:** 1 College of Pharmaceutical Sciences, Zhejiang University, 866 Yuhangtang Road, Hangzhou 310058, China; zhangjl_ra@zju.edu.cn (J.Z.); 22319095@zju.edu.cn (J.M.); 22219139@zju.edu.cn (Y.H.); 2 Institute of Biochemistry, College of Life Sciences, Zhejiang University, 866 Yuhangtang Road, Hangzhou 310058, China; 22407026@zju.edu.cn

**Keywords:** metformin, docetaxel, chemotherapy, administration sequence

## Abstract

**Background:** Chemotherapy has a broad-spectrum anti-tumor effect and is still the core strategy for cancer treatment. However, the side effects caused by its cytotoxicity, the chemoresistance caused by tumor heterogeneity and abnormal microenvironment seriously restrict the efficacy of chemotherapy. Metformin presents the ability to sensitize chemotherapy by interfering with metabolic processes of tumor cells. However, as a dynamic process, metabolic intervention requires a specific time sequence law to optimize its role. **Methods:** Different administration sequences were screened by in vitro experiments to determine the optimal sequence of metformin and docetaxel. The anti-tumor effect of administration sequence in vivo was investigated in mouse models. The therapeutic advantages were comprehensively evaluated by tumor size, weight change, and survival rate. The immunofluorescent staining and transcriptome analysis were performed to study the mechanisms of the sequential administration strategy. **Results:** Compared with the subsequent administration and concurrent administration, pretreatment with metformin exhibited a stronger ability toward cell cycle arrest and tumor inhibition with low-dose docetaxel. Moreover, this pre-administration sequence could enhance the anti-tumor immune responses and prevent postoperative recurrence. **Conclusions:** The optimized chemotherapy sensitization mediated by metabolic intervention required an appropriate administration sequence, which also strengthened the anti-tumor immune responses.

## 1. Introduction

Due to its broad-spectrum anti-tumor effects based on cytotoxicity to rapidly proliferating cells, chemotherapy is still a cornerstone in the management of malignant tumors [1,2,3,4]. However, the mechanisms to inhibit proliferation and induce apoptosis through interfering with DNA replication, suppressing microtubule dynamics or blocking nucleotide metabolism make chemotherapy a double-edged sword, as it also causes great damages to normal tissues [5,6,7,8,9]. Moreover, the tumor heterogeneity and abnormal microenvironment establish chemoresistance via metabolic reprogramming, cell differentiation, drug efflux, drug degradation and DNA damage repair [10,11,12,13]. It prompts dose escalation and multidrug combination, leading to the increased risk of toxic side effects [14,15,16,17]. Therefore, it is critical to develop strategies for chemotherapy sensitization.

Metformin (Met), a classic anti-diabetic agent, has become a promising sensitizer for different cancer treatments, especially for chemotherapy [18,19,20,21]. Because of its metabolic modulation ability, metformin can disturb tumor homeostasis [22,23,24]. Metformin can inhibit the activity of mitochondrial complex I, activating the adenosine 5′-monophosphate-activated protein kinase (AMPK) signal axis, which will suppress anabolic pathways through the mammalian target of the rapamycin (mTOR) pathway [25,26]. In addition, with the downregulation of insulin-like growth factor-1 (IGF-1) signal, metformin can also weaken pro-proliferation networks [27,28,29]. Notably, the ion-trapping effects of chemotherapy caused by acidic tumor microenvironment can be reversed because of the suppression ability to lactate dehydrogenase (LDH) of metformin [30,31,32,33,34]. In a word, metformin could enhance the efficacy of chemotherapy and improve cancer outcomes.

However, the metabolic activity of tumors is a dynamic and continuous process; so, the metabolic intervention caused by metformin is time-dependent [35,36,37]. In order to achieve the optimized sensitization effect, chemotherapy should be involved at the appropriate time, which suggests that a specific administration sequence is required. In addition, the metabolic regulation of metformin has also been proved to modulate the tumor immune microenvironment, resulting in the regulation of immune activities such as T cell memory differentiation and dendritic cell maturation [38,39,40]. But the current study on the immune status of tumor microenvironment after sequential administration of metformin and chemotherapy is not sufficient.

In this article, we evaluated different administration sequences of metformin and docetaxel both in vitro and in vivo to determine an optimal choice. The anti-tumor effect was investigated in mouse models with 4T1 breast cancer. Immunofluorescent staining and transcriptome analysis were performed to study the mechanisms of the sequential administration strategy, especially the influences on cell cycle and immune responses.

## 2. Materials and Methods

### 2.1. Materials

The Cell Counting Kit 8 (cck8), Lyso-Tracker Red and the Cell Cycle and Apoptosis Analysis Kit were purchased from Beyotime Institute of Biotechnology (Shanghai, China). Metformin was purchased from Meilun Biotechnology Co., Ltd. (Dalian, China). FITC-anti-CD3 antibody and other antibodies against cell surface markers for the flow cytometry (fluorescent-activated cell sorting) assay were purchased from eBioscience (San Diego, CA, USA). InVivomAb anti-mouse CD8α (clone 53–67) and anti-mouse CD4 (cloneGK1.5) were purchased from BioXcell (West Lebanon, NH, USA). Docetaxel was purchased from Nanfang pharmaceutcal Co., Ltd. (Fuzhou, Fujian, China), and medium-chain triglyceride (MCT), soyabean lecithin (S100), and Vitamin E (VE, alpha-tocopheryloxyacetic acid) were obtained from Lipoid Co. (Ludwigshafen, Germany). All the chemicals and solvents were of analytical grade and were used as received.

### 2.2. Cells and Animals

Human breast cancer cell line MCF7 and mouse breast cancer cell line 4T1 were originally obtained from the Institute of Biochemistry and Cell Biology (Shanghai, China), and maintained in high-glucose DMEM (GNM12800, JiNuo Biotechnology Co., Ltd., Hangzhou, Zhejiang, China) supplemented with 10% fetal bovine serum (FBS) (16140071, Gibco life technologies) in a humidified atmosphere containing 5% CO_2_.

Female BALB/c mice (6–8-weeks, 18–20 g) were purchased from Slaccas Experimental Animal Co., Ltd. (Shanghai, China) and housed under standard conditions of animal maintenance at 24 ± 1 °C with an alternate 12 h light–dark cycle. All experimental procedures were conducted according to the protocols approved by the Institutional Animal Care and Use Committee of Zhejiang University.

### 2.3. Preparation of Nanoemulsions

The docetaxel nanoemulsions (DOC nps) were prepared by high-energy emulsification method using high-pressure homogenization (HPH). Briefly, docetaxel was dissolved in a mixed medium of VE, S100, and MCT to form an oil phase, which was further dispersed in an aqueous phase containing sucrose. Finally, oil droplets were dispersed into nanoscale-sized particles using the HPH method, and then the solution was filtered through a 0.45 mm pore-sized membrane, lyophilized and stored at 4 °C for further use.

The blank nanoemulsions (Blank nps) and DiD-labeled nanoemulsions (DiD nps) were prepared in same way. For Blank nps, an oil phase without docetaxel was used; and for DiD nps, an 0.5% DiD oil phase without docetaxel was used.

### 2.4. In Vitro Therapeutic Efficacy of the Sequential Strategy Against Breast Cancer Cells

MCF7 cells were seeded into 96-well culture plate at a density of 5 × 10^3^ cells in 0.1 mL complete medium per well. When the cells reached the logarithmic growth phase, they were treated with different doses of Taxotere^®^ and different administration sequence of 1 mg/mL metformin: for the 1M/2T group, the cells were treated with metformin 6 h before Taxotere^®^; for the 1T/2M group, the cells were treated with metformin 6 h after Taxotere^®^; for the M/T group, the cells were treated with metformin and Taxotere^®^ at the same time; for the Tax group, the cells were treated with Taxotere^®^ alone. Cell viability was monitored 24 h or 48 h after the treatment of Taxotere^®^ by cck8 kit.

MCF or 4T1 cells were seeded into 96-well culture plate at a density of 5 × 10^3^ cells in 0.1 mL complete medium per well. When the cells reached the logarithmic growth phase, they were treated with different doses of DOC nps or metformin. Cell viability was monitored 48 h after the treatment by cck8 kit.

For the study of sequential strategy, MCF or 4T1 cells were treated with DOC nps (10 µg/mL docetaxel for MCF7, 5 µg/mL for docetaxel) and different administration sequences of 1 mg/mL metformin: for the 1M/2D group, the cells were treated with metformin 6 h before DOC nps; for the 1D/2M group, the cells were treated with metformin 6 h after DOC nps; for the M/D group, the cells were treated with metformin and DOC nps at the same time; for the Blank NPS group, the cells were treated with the nanoemulsions prepared by the same method of DOC nps but without docetaxel. Cell viability was monitored 48 h after the DOC nps treatment by cck8 kit.

### 2.5. Cellular Uptake and Distribution

4T1 cells were seeded into a 24-well culture plate at a density of 1 × 10^4^ cells in 0.5 mL complete medium per well and subsequently treated with 5 μL/mL of DiD-labeled nanoemulsions (DiD nps). Metformin was added into the wells (1 mg/mL in the medium) according to the prespecified group. Then, 24 h after DiD nps co-incubation, the cells were harvested.

For the cellular uptake study, the cells were washed twice with PBS and stained with Hoechst 33342 (10 μg/mL) for 30 min at room temperature to visualize the nucleus.

For intracellular distribution study, the cells were washed twice with PBS and received the following operations in sequence: stained with Lyso-Tracker Red (50 nM) for 15 min, treated with 3% glutaraldehyde for 10 min, and stained with Hoechst 33342 (10 μg/mL) for 10 min. The cells were washed with PBS between each operation.

After an extensive wash with PBS, fluorescence pictures were captured by a laser confocal fluorescence microscope (A1R, Nikon, Tokyo, Japan), and further analyzed using graphic processing software ImageJ (v1.52a) to semi-quantitate the fluorescence intensity of DiD.

### 2.6. Cell Cycle and Apoptosis Analysis

4T1 cells were treated with DOC nps (5 μg/mL docetaxel) and metformin (1 mg/mL) separately, simultaneously, or as the prespecified administration sequence. Then, 24 h after Doc nps co-incubation, the cells of every group were harvested and stained with Cell Cycle and Apoptosis Analysis Kit (Beyotime Institute of Biotechnology, Shanghai, China). The results were detected by a flow cytometer (ACEA Novo CyteTM, ACEA Biosciences, Inc., San Diego, CA, USA) and analyzed by FlowJo software (v10.8.1).

### 2.7. In Vivo Anti-Tumor Efficacy of Sequential Strategy in the 4T1 Breast Cancer Mouse Model

4T1 cells (1 × 10^6^) were orthotopic injected under the left breast fat pad of female Balb/c mice to establish the orthotopic breast cancer model. When the tumor volume reached 50 mm^3^, the mice were randomly divided into 5 groups (5 mice for each group): Saline, Free Met (5 mg/kg), DOC nps, M/D (treated with 5 mg/kg metformin and DOC nps at the same time), 1M/2D (treated with 5 mg/kg metformin 6 h before DOC nps).

Within 21 days, the mice received intravenous or intra-tumoral administration every 7 days, as described in the schematic diagram of the therapeutic regimen. The tumor volume was recorded and calculated with the following formula: width × length × height × π/6. At the end of the experiment or when the tumors were larger than 1500 mm^3^ or the body weight loss was >20%, the mice were sacrificed. The tumors and organs of the mice were collected.

For the intravenous administration of metformin, the dosage of docetaxel was 8 mg/kg (i.v.). And for the intra-tumoral administration of metformin, the dosage of docetaxel was 4 mg/kg (i.v.). A certain number of tumors in each group were made into a single-cell suspension, and stained with fluorescence-labeled antibodies following the manufacturer’s instruction for flow cytometry. All tissue sections’ H&E staining and immunofluorescence staining for animal experiments were authorized by Haoke Biotechnology Co., Ltd. (Hangzhou, China).

RNA sequencing and library construction were authorized by Lianchuan Biotechnology Co., Ltd. (Hangzhou, China). The RNA-seq data were subjected to quality control analysis by FastQC (v0.12.1) and MultiQC (v1.15). The sequencing reads were then aligned to the reference genome UCSC (mm10) using Hisat2 (v2.2.1), and the alignment results were quantified with HTseq (v2.0.5). Differential analysis was conducted on the quantification results using edgR (v4.2.2), and GO (Gene Ontology) analysis was performed based on org.Mm.eg.db (v3.19.1) using ClusterProfiler (v4.12.6). The R package ggplot2 (v3.5.1) was used for data visualization.

### 2.8. Statistical Analysis

Quantitative data are expressed as the mean ± S.D. When only two groups were compared, a paired *t* test was used. And an evaluation of significance was performed using a one-way ANOVA when more than two groups were compared. All statistical analyses were conducted using GraphPad Prism 8 software.

## 3. Results and Discussion

### 3.1. The Administration Sequence of Metformin Can Affect the Tumor Sensitivity to Chemotherapy

In an in vitro experiment using MCF7 cells (a human breast cancer cell line), we chose docetaxel as the chemotherapy agent. Due to the poor water solubility of docetaxel, Taxotere^®^ (Tax), a commercially available free docetaxel injection, was used, because it provided a suitable solvent in which to dissolve docetaxel. And we set three different administration sequences for study: 1M/2T, treated with metformin 6 h before Tax; 1T/2M, treated with metformin 6 h after Tax; M/T, treated with metformin and Tax simultaneously.

**Figure 1:**
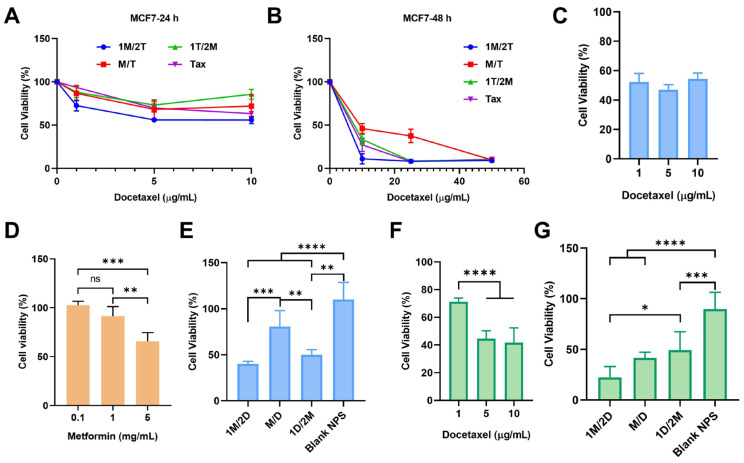
The cell viability of MCF7 cells 24 h (**A**) or 48 h (**B**) after the co-incubation of different concentrations of Tax with the different administration sequences of metformin, *n* = 3. (**C**) The cell viability of MCF7 cells treated with different concentrations of DOC nps at 48 h, *n* = 3. (**D**) The cell viability of MCF7 cells treated with different concentrations of metformin at 48 h, *n* = 4. (**E**) The cell viability of MCF7 cells 48 h after the co-incubation of 10 µg/mL docetaxel with the different administration sequences of 1 mg/mL metformin, *n* = 6. (**F**) The cell viability of 4T1 cells treated with different concentration of DOC nps at 48 h, *n* = 5. (**G**) The cell viability of 4T1 cells 48 h after the co-incubation of 10 µg/mL docetaxel with the different administration sequences of 1 mg/mL metformin, *n* = 6. Tax: treated with Taxotere^®^ alone; 1M/2T: treated with metformin 6 h before Taxotere^®^; 1T/2M: treated with metformin 6 h after Taxotere^®^; M/T: treated with metformin and Taxotere^®^ simultaneously; 1M/2D: treated with metformin 6 h before DOC nps; 1D/2M: treated with metformin 6 h after DOC nps; M/D: treated with metformin and DOC nps simultaneously. Quantitative data are expressed as the mean ± S.D., and one-way ANOVA method was used for statistical analysis. * *p* < 0.05, ** *p* < 0.01, *** *p* < 0.001, **** *p* < 0.0001, ns: no significance.

It was found that the administration sequence of metformin had a significant effect on the function of docetaxel. Compared with the treatment of Tax alone, 1T/2M and M/T, 1M/2T presented the strongest inhibitory effect on tumor cell growth, especially at low docetaxel doses (Figure 1A,B). This suggested that the prior metformin treatment could enhance the sensitivity of tumor cells to chemotherapy.

Nano-particles’ platforms can effectively overcome the solubility problem of hydrophobic drugs and enhance their in vivo therapeutic efficacy. Since the docetaxel nanoemulsions (DOC nps) developed by our laboratory have been fully studied [41,42] and are under a phase I clinical trial (CXHL2200410), we repeated the above experiment with the use of DOC nps. Based on single-drug co-incubation experiments, we selected 10 µg/mL docetaxel for this study (Figure 1C). And it was also verified that 1 mg/mL metformin would not cause significant toxicity (Figure 1D). The results showed that the 1M/2D administration sequence could make DOC nps more toxic to MCF7 cells (Figure 1E). The same phenomenon could be observed on 4T1 cells (a murine breast cancer cell line) treated with 5 µg/mL docetaxel (Figure 1F,G). Moreover, due to the higher sensitivity of 4T1 cells to DOC nps, the enhancement of 1M/2D on chemotherapy was further amplified. At a lower dose of DOC nps, the proliferation of 4T1 cells was significantly inhibited.

The in vitro experiments indicated that metformin-induced sensitization to chemotherapy required a certain administration sequence. This chemosensitization could improve the efficacy and reduce the dose of docetaxel, which might avoid some side effects.

### 3.2. The 1M/2D Administration Sequence Enhanced the Effect of Docetaxel on Cell Cycle Arrest

**Figure 2:**
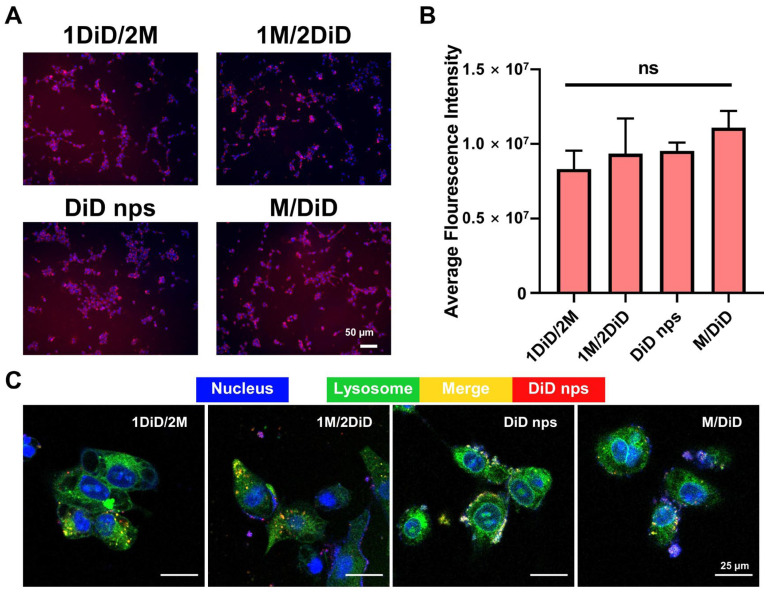
(**A**) The representative fluorescence images of 4T1 cells co-incubating with DiD nps after 24 h. (**B**) Semi-quantitative results obtained by ImageJ (v1.52a) software, *n* = 3. (**C**) The representative fluorescence images of intracellular distribution. DiD nps: DiD-labeled nanoemulsions; 1M/2DiD: treated with metformin 6 h before DiD nps; 1DiD/2M: treated with metformin 6 h after DiD nps; M/DiD: treated with metformin and DiD nps simultaneously. Quantitative data are expressed as the mean ± S.D., and one-way ANOVA method was used for statistical analysis, ns: no significance.

The metabolic regulation induced by metformin may influence the transport function of cells. To eliminate the possibility that enhanced cytotoxicity was due to altered nanoemulsions uptake rather than true chemosensitization, we conducted further investigations into the uptake of nanoemulsions by 4T1 cells. The results demonstrated that the administration sequence of metformin did not affect the cellular uptake of nanoemulsions (Figure 2A,B), and the nanoemulsions were distributed in lysosomes (Figure 2C). Accordingly, we concluded that the enhanced inhibitory effect on tumor cell growth observed in the 1M/2D administration sequence was not attributable to variations in intracellular drug concentration.

**Figure 3:**
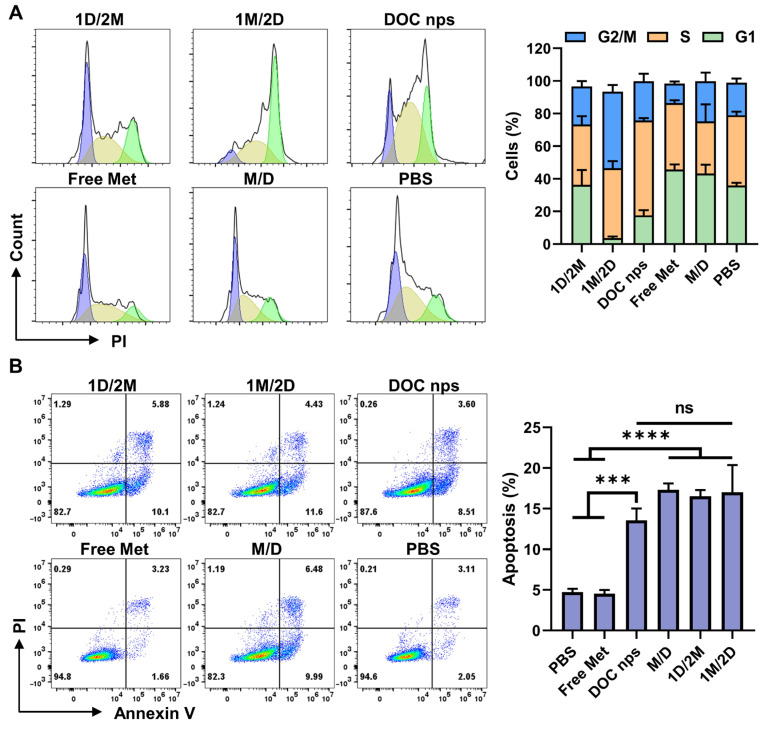
(**A**) The flow cytometry results of cell cycle calculated by FlowJo (v10.8.1) software and its statistical analysis, *n* = 4. (**B**) The flow cytometry results of cell apoptosis and the statistical analysis of total apoptosis (viable and non-viable apoptotic cells), *n* = 3. DOC nps: docetaxel nanoemulsions; Met: metformin; 1M/2D: treated with metformin 6 h before DOC nps; 1D/2M: treated with metformin 6 h after DOC nps; M/D: treated with metformin and DOC nps simultaneously. Quantitative data are expressed as the mean ± S.D., and one-way ANOVA method was used for statistical analysis. *** *p* < 0.001, **** *p* < 0.0001, ns: no significance.

Docetaxel is a taxane anti-tumor agent, which can bind to free tubulin and then promote the assembly of tubulin into stable microtubules to inhibit their disassembly, thereby suppressing cell mitosis. Through the flow cytometry analysis of cell cycle, the 1M/2D treatment led to a significant arrest of 4T1 cells in G2/M phase after 24 h of co-incubation with DOC nps (Figure 3A), which aligned with the pharmacological effects of docetaxel. Meanwhile, the combination of metformin did not enhance cell apoptosis (Figure 3B). Accordingly, we proposed that 1M/2D primarily achieved a pronounced inhibitory effect on tumor cells through synergy with docetaxel’s stabilization of microtubules, but not by inducing apoptosis directly.

### 3.3. The In Vivo Therapeutic Effects of DOC nps with Different i.v. Administration Sequences of Metformin

**Figure 4:**
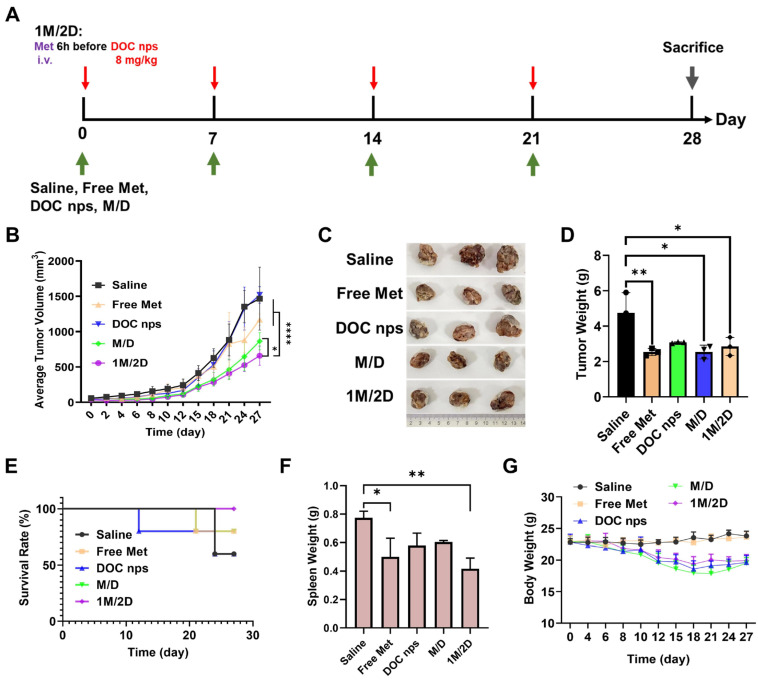
(**A**) Schematic diagram of therapeutic regimen. (**B**) Average tumor volume of each group, *n* = 5. (**C**) Pictures of tumors at day 28. (**D**) Tumor weight of each group, *n* = 5. (**E**) Survival rate of each group, *n* = 5. (**F**) Spleen weight of each group, *n* = 5. (**G**) Body weight of each group, *n* = 5. DOC nps: docetaxel nanoemulsions; Met: metformin; 1M/2D: treated with metformin 6 h before DOC nps; M/D: treated with metformin and DOC nps simultaneously. Quantitative data are expressed as the mean ± S.D., two-way ANOVA method was used for statistical analysis of (**B**), and one-way ANOVA method was used for statistical analysis of (**D**,**F**). * *p* < 0.05, ** *p* < 0.01, **** *p* < 0.0001.

The effect of metformin administration sequence on the efficacy of DOC nps was investigated in 4T1 mouse tumor models, in which metformin was administered intravenously (Figure 4A). The 1M/2D treatment could only slow tumor growth but did not inhibit tumor progression or prolong survival time (Figure 4B–E). After two cycles of treatment, the 1M/2D group showed significantly increased tumor growth, which indicated that the progress of tumor was out of control at this time.

However, the 1M/2D treatment could alleviate splenomegaly, which was a common phenomenon in the 4T1 tumor model (Figure 4F). The mice in all groups that received chemotherapy showed a downward trend in body weight at the later stage, which might be an indication of excessive doses of DOC nps or poor physical condition due to tumor growth (Figure 4G).

### 3.4. The In Vivo Therapeutic Effects of DOC nps with Different Intra-Tumoral Administration Sequences of Metformin

**Figure 5:**
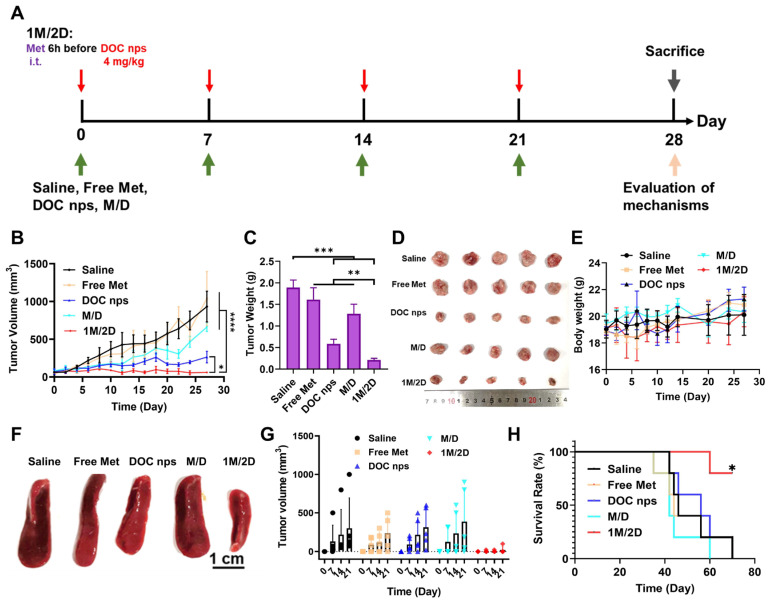
(**A**) Schematic diagram of therapeutic regimen. (**B**) Tumor volume of each group, *n* = 5. (**C**) Tumor weight of each group, *n* = 5. (**D**) Pictures of tumors at day 28. (**E**) Body weight of each group, *n* = 5. (**F**) Representative spleen pictures of each group. (**G**) Tumor recurrence after surgery in each group of mice, *n* = 5. (**H**) Survival rate of each group, *n* = 5. DOC nps: docetaxel nanoemulsions; Met: metformin; 1M/2D: treated with metformin 6 h before DOC nps; M/D: treated with metformin and DOC nps simultaneously. Quantitative data are expressed as the mean ± S.D., two-way ANOVA method was used for statistical analysis of (**B**), one-way ANOVA method was used for statistical analysis of (**C**), and Log-rank (Mantel-Cox) test was used for statistical analysis of (**H**). * *p* < 0.05, ** *p* < 0.01, *** *p* < 0.001, **** *p* < 0.0001.

The results above suggested that the increased sensitivity to chemotherapy mediated by 1M/2D administration might require a certain amount of metformin accumulation in tumor tissues, which could not be achieved by intravenous administration. Therefore, we changed the form of metformin administration to intra-tumoral injection (Figure 5A). In addition, the dose of DOC nps was reduced to avoid toxicity to mice at the later stage.

**Figure 6:**
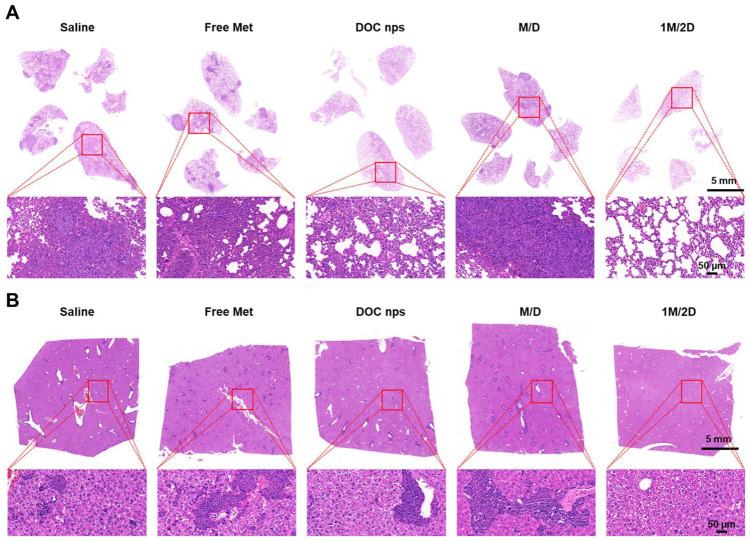
Representative H&E-stained images of lung (**A**) and liver (**B**) from each group of mice. DOC nps: docetaxel nanoemulsions; Met: metformin; 1M/2D: treated with metformin 6 h before DOC nps; M/D: treated with metformin and DOC nps simultaneously.

During the four cycles of treatment, the 1M/2D group demonstrated a significant inhibitory effect on tumor growth (Figure 5B–D). Moreover, due to the reduced dose of DOC nps, the body weight of mice did not show a downward trend throughout the treatment process (Figure 5E). Similarly, the spleens of mice that received 1M/2D treatment did not exhibit obvious splenomegaly (Figure 5F), and there were no obvious tumor metastases in lungs and livers (Figure 6). On day 28 after the first treatment, we performed surgery on mice to remove tumors to simulate postoperative recurrence. During the 21 days after surgery, the recurrence in the 1M/2D treatment group was effectively suppressed (Figure 5G). Throughout the treatment and postoperative observation period, the mice in the 1M/2D group showed the longest survival (Figure 5H).

**Figure 7:**
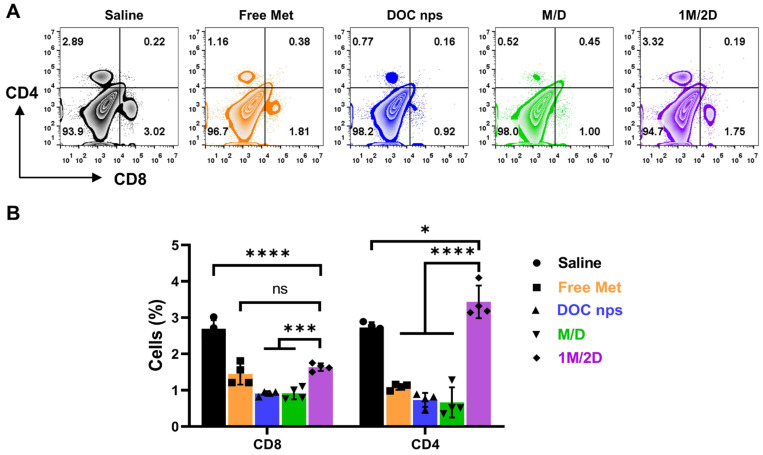
Flow cytometry results (**A**) and statistical analysis (**B**) of tumor tissues from each group, *n* = 4. DOC nps: docetaxel nanoemulsions; Met: metformin; 1M/2D: treated with metformin 6 h before DOC nps; M/D: treated with metformin and DOC nps simultaneously. Quantitative data are expressed as the mean ± S.D, and one-way ANOVA method was used for statistical analysis. * *p* < 0.05, *** *p* < 0.001, **** *p* < 0.0001, ns: no significance.

The markedly different spleen status and tumor recurrence suggested that the immune response to tumor was altered. Therefore, we examined the CD4^+^ and CD8^+^ T cell populations in tumor tissues by flow cytometry. The CD8^+^ T cell population in the 1M/2D group was slightly higher than that in the DOC nps and M/D groups and lower than that in the Saline group (Figure 7). However, its CD4^+^ T cell population was significantly higher than that of the other four groups.

**Figure 8:**
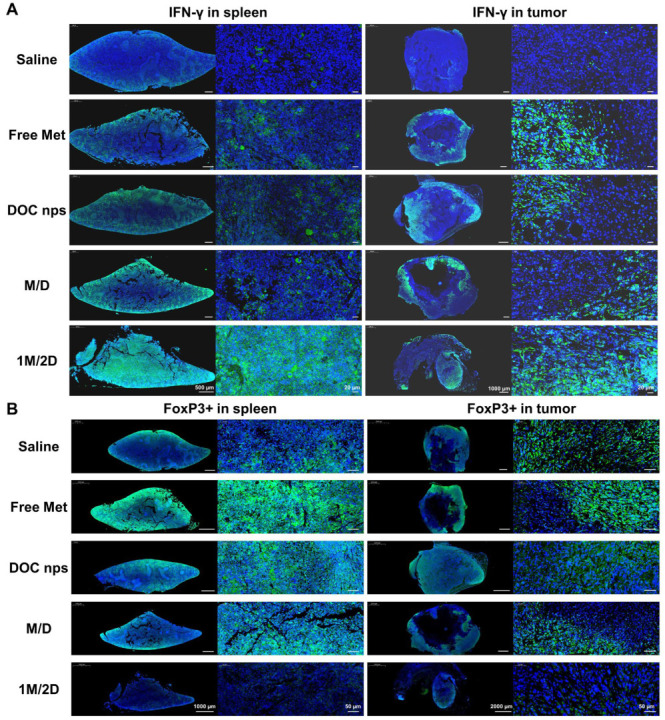
Representative images of IFN-γ (**A**) and FoxP3 (**B**) immunofluorescence staining of spleens and tumors of mice, left: panoramic scan, right: local magnification. DOC nps: docetaxel nanoemulsions; Met: metformin; 1M/2D: treated with metformin 6 h before DOC nps; M/D: treated with metformin and DOC nps simultaneously.

This finding suggested that the 1M/2D treatment was able to influence the T cell population within the tumor microenvironment. To gain deeper insights into this effect, we conducted immunofluorescence staining on spleen and tumor samples. We focused on two key markers: IFN-γ and FoxP3. IFN-γ is an important indicator of anti-tumor immune responses, whose increase suggests enhanced activity of effector T cells. And FoxP3 is expressed by regulatory T cells (Tregs), which can contribute to an immunosuppressive microenvironment. Compared to other groups, the spleens and tumors of the 1M/2D group showed an extremely low level of FoxP3 while displaying an increased IFN-γ level (Figure 8). The balance between pro-inflammatory and immunosuppressive factors within the tumor microenvironment is crucial for the outcome of cancer treatments. By modulating this balance, the 1M/2D treatment appears to create a more favorable environment for anti-tumor immune responses.

**Figure 9:**
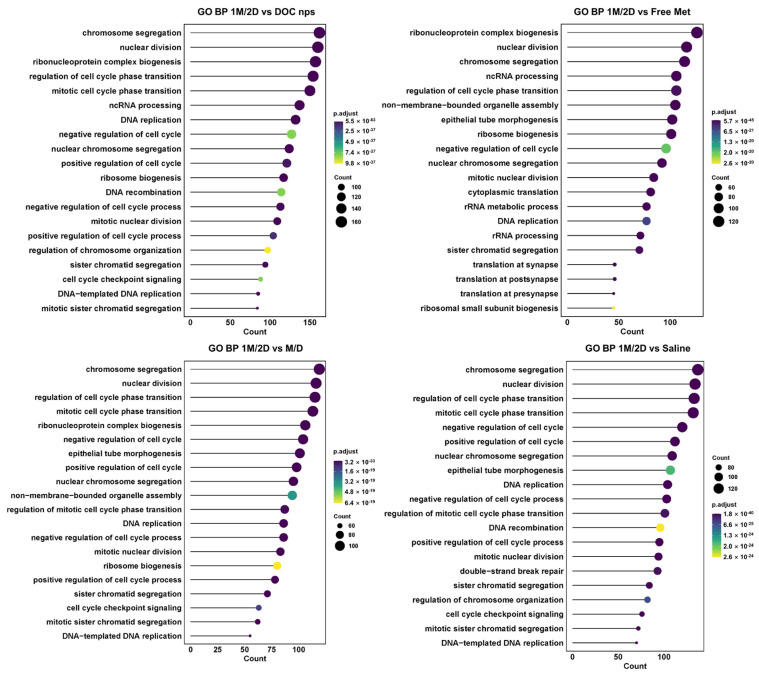
The GO BP enrichment results of downregulated cell cycle-related gene expression between 1M/2D and other groups. DOC nps: docetaxel nanoemulsions; Met: metformin; 1M/2D: treated with metformin 6 h before DOC nps; M/D: treated with metformin and DOC nps simultaneously.

**Figure 10:**
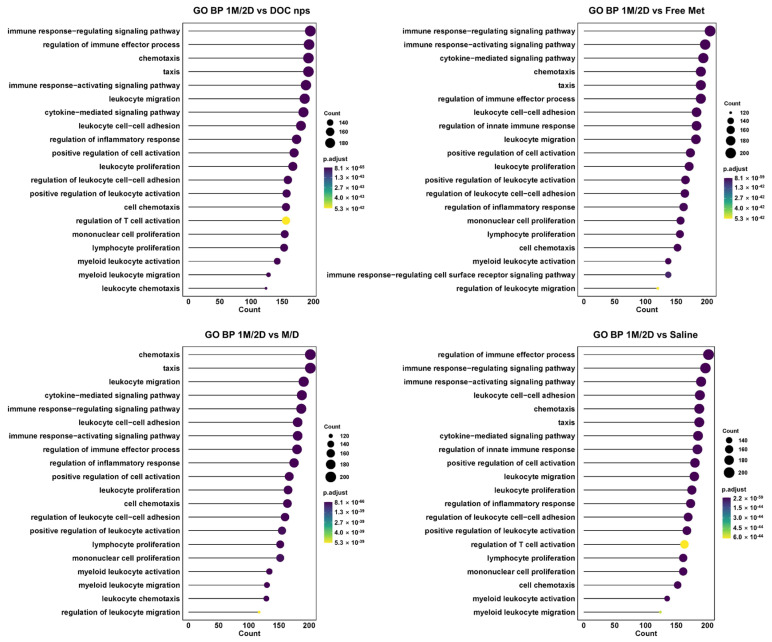
The GO BP enrichment results of upregulated cell communication and immunity-related gene expression between 1M/2D and other groups. DOC nps: docetaxel nanoemulsions; Met: metformin; 1M/2D: treated with metformin 6 h before DOC nps; M/D: treated with metformin and DOC nps simultaneously.

Considering the metabolic intervention of metformin would cause a comprehensive effect rather than a few cytokines, we collected tumor tissues for transcriptome analysis to investigate the changes in cell activities. The Gene Ontology analysis of the biological process (GO BP) demonstrated that the cell cycle of tumors with 1M/2D treatment was obviously disturbed since the associated gene expression was highly downregulated, such as chromosome segregation, nuclear division and cell cycle phase transition (Figure 9). The results of the GO BP also indicated that, compared to other administration sequences, 1M/2D could induce stronger immune responses in tumors, accompanied by the upregulation of cell communication and immunity-related genes, such as immune effector process, chemotaxis, activating signaling pathway and inflammatory response (Figure 10).

The synergistic effect of co-delivering metformin has been studied in many research studies [20,43]. This article reveals that the pretreatment of metformin 6 h before docetaxel nanoemulsions’ administration can significantly optimize its cell cycle arrest and tumor suppression efficacy. These findings underscore the critical importance of the administration sequence in metabolic-intervention-induced chemosensitization. It was reported that treatment with metformin for 48 h followed by withdrawal for 24 h could synchronize cells to enter the S phase, which enhances the damage caused by cisplatin administrated on testicular germ cell tumors after metformin withdrawal [44]. Notably, this sensitization principle extends beyond chemotherapy. For instance, 1 h pretreatment of arginine could effectively disrupt intracellular NAD^+^ homeostasis, compromising cellular DNA repair capacity and consequently enhancing radiosensitivity in brain metastases [45]. Our study may provide a reference for metformin-associated chemotherapy. However, the sequencing administration should be designed based on tumor types, the targets of metabolic intervention and the mechanisms of sensitized therapies.

Furthermore, the chemosensitization with intra-tumoral administration of metformin was stronger as compared to that of *i.v.* metformin. These results indicate that the metabolic intervention requires high drug levels within the tumor. These levels are not achieved by *i.v.* administration of the free drug because it does not have tumor-targeting ability and blood perfusion is usually poor in tumor tissue [46]. Although intra-tumoral administration can be applied for superficial tumors and some deeper tumors with the combination of imaging navigation and endoscopic techniques, its clinical application is still limited. Thus, targeted systems would be a better option for drug delivery to the tumor. Various tumor-targeted delivery systems of metformin, such as liposomes, drug conjugates and cell-derived microparticles, have already been reported [47,48,49,50]. Considering the dynamic nature of tumor metabolism and potential homeostatic recovery mechanisms, transient metabolic interventions may yield suboptimal outcomes [51,52]. This can be solved by the development of sustained-release formulations, which can maintain therapeutic metabolic modulation over the treatment duration.

Cellular metabolism constitutes the fundamental basis of biological functions. Recent advances in research on tumor carbon and nitrogen metabolic pathways have gained increasing interest in oncology [53,54]. The clinical availability of diverse drugs for metabolic modulation presents promising opportunities for developing metabolism-based synergetic therapies. As there is no urgent need for the development of new metabolic drugs, optimizing the administration sequence to further amplify its therapeutic effects seems to be a reasonable and practical way to improve the existing clinical treatment protocols. According to the in vivo experiments, the optimized administration sequence can strengthen tumor inhibition, lower chemotherapy doses and enhance immune responses. We speculate that this strategy might be able to reduce the toxicity of chemotherapy for patients, decrease the incidence of complications, and improve the life quality, especially for those undergoing long-term chemotherapy.

Some possible treatment-related issues should also be taken into consideration. The adverse events that are associated with both metformin and chemotherapy, such as gastrointestinal reaction, may be exacerbated due to combination therapy [55,56]. While metformin may enhance chemotherapy efficacy by enhancing anti-tumor immunity, it could simultaneously attenuate normal immune function by suppressing immune cell metabolism. Metformin clearance is dependent on renal excretion. The nephrotoxicity induced by chemotherapy may facilitate drug accumulation, establishing a vicious cycle of renal damage and lactic acidosis [57]. The heterogeneity and high mutability of tumors may weaken the effects of sequencing strategy. The metabolic-related complications and the emergence of metformin resistance can bring some unexpected risks. Accordingly, further in-depth studies are needed.

Our proposed sequencing strategy may provide valuable insights for addressing current challenges in conventional chemotherapy regimens, particularly in overcoming drug resistance and minimizing systemic toxicity.

## 4. Conclusions

This study systematically investigated the therapeutic potential of administration sequence of metformin and DOC nps. The in vitro evaluations indicated that the 1M/2D treatment could significantly enhance the inhibition on tumor proliferation. This chemosensitization effect was induced by magnifying the cell cycle arrest of docetaxel, which was not related to cellular uptake or cell apoptosis. With reduced docetaxel dosage and intra-tumoral administration of metformin, the 1M/2D treatment presented remarkable therapeutic effects in 4T1 tumor-bearing mice, leading to limited tumor progression, prolonged survival, and enhanced metastasis inhibition. The investigations of immunity demonstrated that the 1M/2D treatment could strengthen anti-tumor immune responses, which decreased postoperative recurrence rates. The obtained results also revealed the necessity to improve the tumor-targeted delivery of metformin. Therefore, we will focus on developing co-encapsulated platforms with controlled-release profiles to optimize spatiotemporal delivery in future research.

## Data Availability

The data are contained within this article.
